# Assessment of two different types of bias affecting the results of outcome-based evaluation in undergraduate medical education

**DOI:** 10.1186/1472-6920-14-149

**Published:** 2014-07-21

**Authors:** Sarah Schiekirka, Sven Anders, Tobias Raupach

**Affiliations:** 1 Department of Cardiology and Pneumology, University Hospital Göttingen, Göttingen, Germany; 2 Study Deanery of Göttingen Medical School, Göttingen, Germany; 3 Department of Legal Medicine, University Medical Centre Hamburg Eppendorf, Hamburg, Germany; 4 Department of Clinical, Educational and Health Psychology, University College London, London, UK

**Keywords:** Undergraduate medical education, Evaluation, Learning outcome, Response shift bias

## Abstract

**Background:**

Estimating learning outcome from comparative student self-ratings is a reliable and valid method to identify specific strengths and shortcomings in undergraduate medical curricula. However, requiring students to complete two evaluation forms (i.e. one before and one after teaching) might adversely affect response rates. Alternatively, students could be asked to rate their initial performance level retrospectively. This approach might threaten the validity of results due to response shift or effort justification bias.

**Methods:**

Two consecutive cohorts of medical students enrolled in a six-week cardio-respiratory module were enrolled in this study. In both cohorts, performance gain was estimated for 33 specific learning objectives. In the first cohort, outcomes calculated from ratings provided before (pretest) and after (posttest) teaching were compared to outcomes derived from comparative self-ratings collected after teaching only (thentest and posttest). In the second cohort, only thentests and posttests were used to calculate outcomes, but data collection tools differed with regard to item presentation. In one group, thentest and posttest ratings were obtained sequentially on separate forms while in the other, both ratings were obtained simultaneously for each learning objective.

**Results:**

Using thentest ratings to calculate performance gain produced slightly higher values than using true pretest ratings. Direct comparison of then- and posttest ratings also yielded slightly higher performance gain than sequential ratings, but this effect was negligibly small.

**Conclusions:**

Given the small effect sizes, using thentests appears to be equivalent to using true pretest ratings. Item presentation in the posttest does not significantly impact on results.

## Background

Programme evaluation in medical education should be multi-dimensional, combining subjective and objective data to gather comprehensive information on teaching processes and learning outcome [1,2]. So far, few evaluation tools directly assess learning outcome for specific learning objectives. We have recently developed an outcome-based evaluation tool which is based on student self-assessments collected at the beginning and the end of a course. By taking into account pre-post differences and controlling for initial performance levels, it facilitates an appraisal of performance gain for specific learning objectives. This approach has been shown to be superior to measuring plain pre-post differences or effect sizes: Unlike performance gain, effect sizes are sensitive to differences in standard deviations between groups, and learning outcome may be underestimated if initial performance levels are high [3]. Evaluation results obtained with the novel tool appear to be independent of traditional evaluation parameters, e.g. ratings of student satisfaction with courses [4]. A recent validation study using objective measures of student performance established good criterion validity of the tool [5]. However, the need to collect data both at the beginning and the end of a course from the same student group poses a practical challenge and is likely to adversely affect response rates [6]. Given that students prefer evaluation activities to be kept to a minimum [7], higher response rates may be expected if self-ratings of initial and final performance levels are collected at one single time-point, e.g. the end of a course.

Research dating back to the 1970s indicated that asking individuals to retrospectively assess their past performance level in a so-called ‘thentest’ is likely to have a considerable impact on change scores reflecting differences between true pretests and posttests [8]. Various explanations for the observed differences between true pretest and retrospective thentest ratings (both targeting the same point in time) have been suggested. According to one theory, students self-rating their performance levels at the beginning of a course (true pretest) might overestimate their abilities as they lack a complete understanding of the complexity of the content taught. Following exposure to teaching, this understanding is likely to be more complete, resulting in less favourable thentest ratings of initial performance levels. In other words, exposure to teaching changes a student’s internal standards to benchmark their own performance. This effect known as ‘response shift bias’ (RSB) has been investigated extensively [9]. It has been argued that change scores calculated from thentest and posttest ratings are more valid than scores calculated from true pretests and posttests [8]. However, other studies have yielded evidence of artificial (i.e. invalid) inflation of change scores when using thentests: In one trial [10], students were asked to self-rate both their initial and their final performance levels at the end of a course. Results indicated a considerable improvement – however, the course was actually ineffective. One potential explanation for this phenomenon is that course participants assumed to have learned something in a course that was meant to be effective (implicit theories of change, [11]). This ‘expected change’ was reflected in greater differences between thentest and posttest ratings. Likewise, students who have invested considerable resources in completing a course assignment might be prone to overestimating their learning outcome; this effect is called ‘effort justification bias’ [8].

Unlike the impact of response shift bias on thentest ratings, the effect of such implicit theories of change does not require a recalibration of internal standards. In both cases (response shift and implicit theories of change), the net effect is an inflation of change scores. While response shift is usually thought to increase the validity of evaluation results [12-18], effects related to implicit theories of change are generally thought to decrease validity.

Whichever the cause, any difference between true pretest and thentest ratings is likely to affect the results of the outcome-based evaluation tool as it uses student self-assessments to estimate performance gain. So far, the effect of using thentest ratings instead of true pretest ratings on performance gain has not been investigated. While the impact of response shift bias and implicit theories of change on single ratings and change scores has been researched in great depth, no previous study has evaluated their impact on estimated performance gain.

A second issue that has rarely been discussed in this context is the mode of data collection. Students self-rating their performance levels at the end of a course are usually asked to provide thentest ratings for all learning objectives first before completing a second questionnaire eliciting posttest ratings for all learning objectives. In this scenario, response shift – if present – might lead to an increase in change scores by prompting students to provide particularly bad ratings of their initial performance level. However, other forms of bias (e.g., implicit theories of change, effort justification bias) are less likely to confound results unless students are encouraged to directly compare their thentest and posttest ratings. Such comparison is less likely to occur if the two questionnaires are completed sequentially. An alternative approach could be to present students with one list of all learning objectives and ask them to provide both thentest and posttest ratings for each of them. As this will produce a ‘visual change score’, ratings might be influenced by implicit theories of change in that students believing to have learned a lot will provide inflated change scores. In this scenario, both response shift and implicit theories of change could be at work. A better understanding of these processes would be helpful to guide interpretation of evaluation results obtained with the outcome-based tool.

The aim of this study was to answer the following research questions:

(1) What is the impact of using thentest ratings instead of true pretest ratings on performance gain as calculated with the novel evaluation tool?

(2) How does the design of the data collection tool (i.e. sequential data collection vs. direct comparison of thentest and posttest ratings for specific learning objectives) impact on performance gain?

Based on the theoretical considerations outlined above, we derived the following hypotheses:

(a) Thentest ratings are less positive than true pretest ratings, thus producing higher performance gain values.

(b) Direct comparisons of thentest and posttest ratings for specific learning objectives yield higher performance gain values than sequential data collection, and this difference may reflect the bias introduced by implicit theories of change.

As neither RSB nor implicit theories of change should alter posttest ratings, we also hypothesised that differences between performance gain derived from the two different data collection tools would be due to a change in thentest ratings but not posttest ratings.

## Methods

### Research setting

The six-year undergraduate medical curriculum at our institution comprises two pre-clinical and three clinical years, followed by a practice year. The clinical part of the curriculum has a modular structure: There are 21 modules lasting two to seven weeks each; the sequence of modules is identical for all students. Thus, all students take part in a six-week, interdisciplinary cardio-respiratory module at the beginning of year four. Two consecutive cohorts of students enrolled in this module (summer 2011 and summer 2012) were invited to participate in two separate studies designed to address the two research questions. Students received an e-mail outlining the study rationale four weeks before the beginning of the module.

### Study design

**Figure 1:**
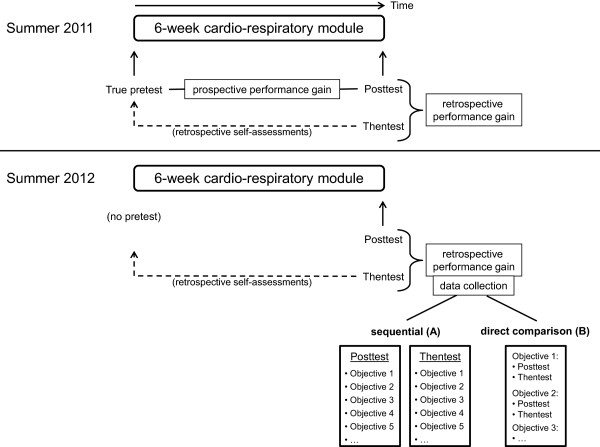
Study design (see text for details).

The outline of the study design is displayed in Figure 1.

Study 1: In order to address research question 1, study participants in summer 2011 were asked to provide self-assessments of their current performance level regarding 33 learning objectives on the first day of the cardio-respiratory module (true pretest). On the second day of the last week of the module, students provided self-assessments of their current performance level regarding the same 33 learning objectives (posttest) and, subsequently, retrospective ratings of their initial performance level regarding these objectives (thentest). Thus, posttest and thentest ratings were collected sequentially in this cohort.

Study 2: In order to address research question 2, no data collection was necessary at the beginning of the module in summer 2012. As in study 1, posttest and thentest ratings were collected during the last week of the module. However, study participants were stratified by sex and prior exam results and subsequently randomised to two different data collection methods: Students in group A (sequential data collection as in cohort 1) provided posttest ratings regarding the 33 learning objectives printed on one list and, subsequently, thentest ratings regarding the same objectives on a second list. Students in group B (direct comparison) received only one list of learning objectives and were asked to provide both posttest and thentest ratings for each objective. Thus, differences between posttest and thentest ratings for specific learning objectives were instantly apparent to students in group B but not to students in group A. Students in group A were instructed not to actively compare their posttest and thentest ratings. Students in both groups completed the study questionnaires on the same day and at the same time but in different lecture theatres.

In both cohorts, basic demographic information (i.e., gender and age) was collected at study entry. In order to assess the comparability of the two cohorts, we also obtained student scores achieved in the summative end-of-module examination consisting of 69 multiple choice questions assessing knowledge on the diagnosis and treatment of cardiovascular and respiratory disease. We used unique student identifier codes to facilitate matching of self-assessments and end-of-module examination data as well as to randomize study participants in summer 2012. Only the study coordinator (TR) had access to these codes, and all codes were deleted from the dataset prior to statistical analysis.

This study was an extension to the recently published validation study [5]. That study was reviewed by the institutional review board of Göttingen Medical School (application number 27/3/11). Ethical approval was waived because the study protocol was not deemed to represent biomedical or epidemiological research. We made every effort to comply with data protection rules. Study participation was voluntary, and all participants signed an informed consent form before entering the study.

### Calculation of performance gain and unit of analysis

The statements used for self-assessments were derived from the institution’s Catalogue of Specific Learning Objectives (e.g., ‘I know the five principal risk factors for the development of coronary artery disease’.). The same statements had been used in a previous study assessing criterion validity of the evaluation tool [5]. Student ratings were provided on 6-point scales anchored at 1 (‘fully agree’) and 6 (‘completely disagree’).

In a critical appraisal of research involving self-assessments, Lam [19] demonstrated how the unit of analysis impacts on evaluation results: Calculations can either be performed at the individual level (i.e. using data obtained from individual students as singular data points) or at the group level (i.e. using aggregated data by computing group means). The unit of analysis (individual or group) needs to be taken into account when reporting findings from evaluation studies since performance differences tend to be greater as the unit of analysis increases [20]. We have previously shown that the outcome-based evaluation tool requires data to be analysed on the group-level in order to render valid results [5]. Thus, aggregated data derived from the entire student cohort (summer 2011) or randomisation groups (summer 2012) were used in this study. Mean student self-assessments were used to calculate performance gain according to the following formulas:

$$\begin{array}{l} \text{Prospective}\;\text{Performance}\;\text{Gain}\;\left[\%\right]=\frac{\mu_{\text{pretest}}-\mu_{\text{posttest}}}{\mu_{\text{pretest}}-1}\times 100\\ \text{Retrospective}\;\text{Performance}\;\text{Gain}\;\left[\%\right]=\frac{\mu_{\text{thentest}}-\mu_{\text{posttest}}}{\mu_{\text{thentest}}-1}\times 100 \end{array}$$

### Statistical analysis

Differences between groups were assessed by χ^2^ tests (dichotomous variables) and t-Tests (continuous variables). Differences between true pretest and thentest ratings (summer 2011) as well as between the two data collection tools (summer 2012) were also expressed as Cohen’s d [21] with values of 0.2 indicating small and values of 0.8 large effects. The specific research questions were addressed as follows:

(1) Effect sizes of the difference in true pretest ratings and thentest ratings of initial performance levels for the 33 learning objectives were displayed as a histogram. Negative effect sizes indicated that thentest ratings were more pessimistic than true pretest ratings; according to our hypothesis and the available literature, we expected most of the 33 effect sizes to be negative. Agreement between prospective and retrospective performance gain values was assessed by means of a Pearson correlation.

(2) Effect sizes of differences between the two methods of data collection (sequential/direct comparison) were calculated for thentest and posttest ratings. These effect sizes were displayed in two separate histograms. In both cases, negative effect sizes indicated that ratings were more pessimistic in group B (direct comparison). Agreement between performance gain values derived from the two different data collection tools was assessed by means of a Pearson correlation.

## Results

### Response rates and sample characteristics

In summer 2011, 115 out of 145 students enrolled in the cardio-respiratory module gave written consent to participate in the study (response rate 79.3%). A total of 32 students did not participate in the second data collection in the last week of the module and had to be excluded, leaving 83 students with complete data available for the analysis (final response rate 57.2%). In summer 2012, 106 out of 133 students enrolled in the module gave written consent to participate (response rate 79.7%). Again, 32 students dropped out of the study; thus, complete data were available for 74 students (final response rate 55.6%). There was no significant difference in the age of study completers between the two cohorts (25.0 ± 2.4 vs. 24.5 ± 2.6; t(155) = 1.118; p = 0.265), but the proportion of females was smaller in summer 2011 (50/83) than in summer 2012 (56/74; χ^2^(1) = 4.249; p = 0.039). Percent scores achieved in the end-of-module examination were similar in the two cohorts (81.0 ± 7.3 vs. 79.6 ± 9.5; t(153) = 1.0789; p = 0.282).

### Comparison between true prospective and retrospective performance gain (summer 2011)

**Figure 2:**
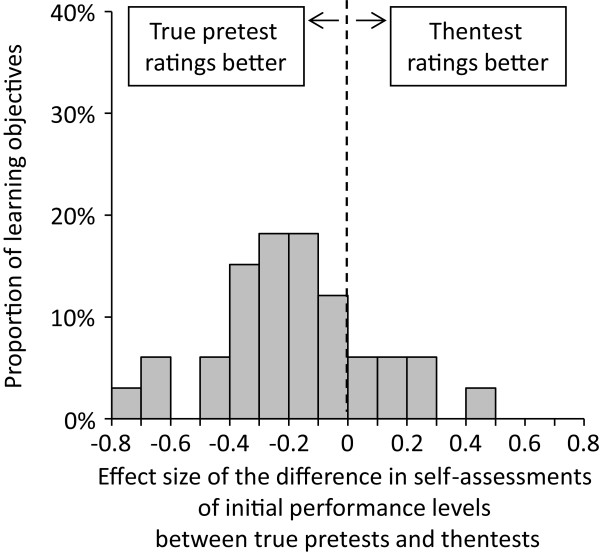
Impact of the time-point of data collection on initial performance level ratings for 33 learning objectives.

**Figure 3:**
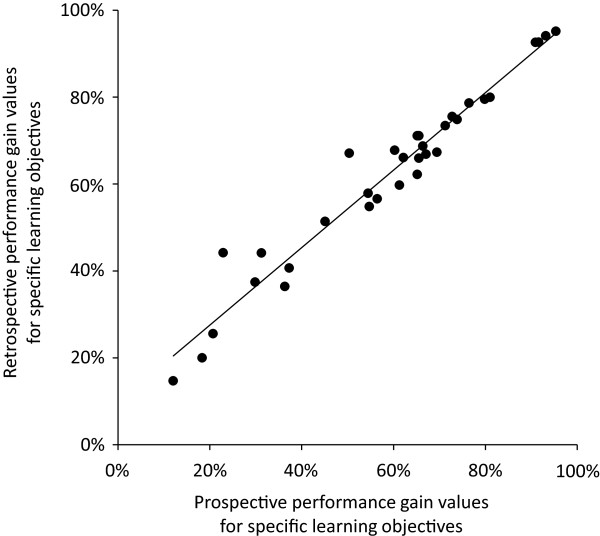
Correlation between prospective and retrospective performance gain values for 33 learning objectives (r = 0.975; p < 0.001).

Mean true pretest ratings for individual learning objectives collected at the beginning of the module ranged from 2.87 to 5.90 with smaller values indicating more positive self-assessments (see Table 1). Thentest ratings collected at the end of the module ranged from 3.23 to 5.74. Effect sizes of the differences between both ratings for each of the 33 learning objectives are displayed in Figure 2. Effect sizes ranged from -0.75 to 0.48 and were negative for 26 out of 33 learning objectives (mean effect size -0.18, indicating a small effect). Prospective performance gain values ranged from 12.0% to 95.3% with a mean gain of 58.9%. Retrospective performance gain values were slightly higher (range: 14.7% to 95.1%; mean: 62.2%). There was a strong and significant correlation between prospective and retrospective performance gain values (r = 0.975; p < 0.001; see Figure 3). In summary, these data supported hypothesis (a).

**Table 1:** Student self-assessments for 33 specific learning objectives (1 = most positive rating; 6 = most negative rating)

**Learning objectives**	**Summer 2011**		**Summer 2012**			
	**Initial performance level**		**Thentest rating**		**Posttest rating**	
	**True pretest (n = 83)**	**Thentest (n = 83)**	**Group A: sequential (n = 42)**	**Group B: direct (n = 32)**	**Group A: sequential (n = 42)**	**Group B: direct (n = 32)**
Acute bronchitis	4.35 ± 1.31	4.75 ± 1.24	5.00 ± 0.99	4.56 ± 1.13	3.21 ± 1.46	3.09 ± 1.06
Pericarditis	5.90 ± 0.30	5.51 ± 1.13	5.50 ± 1.11	5.50 ± 1.27	2.74 ± 1.48	2.34 ± 1.45
Acute arterial occlusion	3.82 ± 1.46	4.13 ± 1.40	4.10 ± 1.30	4.53 ± 1.32	1.74 ± 1.31	1.69 ± 0.93
Acute myocardial infarction	3.14 ± 1.41	3.63 ± 1.62	3.93 ± 1.40	4.41 ± 1.37	1.36 ± 0.53	1.63 ± 1.04
Aortic aneurysm & dissection	5.19 ± 1.38	4.91 ± 1.53	5.40 ± 1.17	5.38 ± 1.21	1.55 ± 0.77	1.47 ± 1.05
Aortic stenosis	4.58 ± 1.35	4.94 ± 1.36	5.22 ± 1.15	5.19 ± 1.15	1.74 ± 0.99	2.00 ± 1.08
Asthma	5.14 ± 1.10	5.31 ± 1.13	5.02 ± 1.33	5.34 ± 1.34	2.05 ± 1.13	2.13 ± 0.98
Atherosclerosis	2.94 ± 1.38	3.39 ± 1.38	3.32 ± 1.29	3.84 ± 1.51	1.79 ± 0.81	1.94 ± 1.22
Chronic bronchitis	4.28 ± 1.48	4.66 ± 1.37	4.56 ± 1.34	4.78 ± 1.29	2.29 ± 1.18	2.03 ± 1.09
COPD	5.42 ± 0.80	5.34 ± 1.23	5.29 ± 1.10	5.34 ± 1.31	1.60 ± 0.94	1.94 ± 1.01
Pulmonary emphysema	5.42 ± 0.68	5.48 ± 1.02	5.29 ± 1.03	5.19 ± 1.42	2.64 ± 1.48	2.31 ± 1.23
Endocarditis	5.55 ± 0.97	5.52 ± 1.17	5.51 ± 1.31	5.66 ± 1.13	2.02 ± 1.39	1.91 ± 1.15
Tetralogy of Fallot	5.18 ± 1.33	5.01 ± 1.61	5.12 ± 1.74	5.38 ± 1.52	1.57 ± 1.35	1.41 ± 1.23
Congestive heart failure	4.59 ± 1.31	5.07 ± 1.34	4.76 ± 1.48	5.41 ± 1.24	1.64 ± 0.79	1.59 ± 1.10
Arterial hypertension	3.58 ± 1.42	4.56 ± 1.21	4.44 ± 1.48	4.69 ± 1.33	3.14 ± 1.49	2.34 ± 1.23
Influenza	2.87 ± 1.44	3.81 ± 1.40	4.27 ± 1.58	4.59 ± 1.48	2.48 ± 1.35	2.47 ± 1.22
Cardiomyopathy	5.73 ± 0.59	5.55 ± 0.93	5.50 ± 1.18	5.59 ± 1.16	2.64 ± 1.21	2.53 ± 1.11
Coronary artery disease	2.93 ± 1.53	3.23 ± 1.32	2.85 ± 1.30	3.63 ± 1.58	1.21 ± 0.47	1.31 ± 1.00
Pulmonary embolism	5.67 ± 0.61	5.68 ± 0.83	5.37 ± 0.99	5.22 ± 1.39	2.45 ± 1.15	2.41 ± 1.16
Mitral regurgitation	4.69 ± 1.22	4.99 ± 1.26	4.90 ± 1.15	5.28 ± 1.05	2.07 ± 0.89	2.25 ± 1.02
Myocarditis	4.96 ± 1.13	5.28 ± 1.03	5.15 ± 1.26	5.44 ± 1.16	2.38 ± 1.06	2.31 ± 0.93
Peripheral artery disease	4.95 ± 1.38	5.21 ± 1.28	4.83 ± 1.47	4.88 ± 1.54	2.17 ± 1.31	2.47 ± 1.61
Patent foramen ovale	5.45 ± 0.94	5.45 ± 1.06	5.46 ± 1.00	5.66 ± 1.13	2.90 ± 1.59	2.69 ± 1.53
Pleural effusion	5.32 ± 0.89	5.33 ± 1.02	5.07 ± 1.37	5.41 ± 1.16	2.69 ± 1.28	2.91 ± 1.25
Pneumonia	4.59 ± 1.43	4.85 ± 1.30	4.98 ± 1.29	5.31 ± 1.12	2.31 ± 1.35	2.41 ± 1.39
Pneumothorax	3.48 ± 1.36	3.99 ± 1.37	4.32 ± 1.46	4.59 ± 1.39	2.33 ± 1.20	2.19 ± 1.15
Pulmonary hypertension	4.49 ± 1.37	5.30 ± 1.01	5.10 ± 1.18	5.22 ± 1.39	3.36 ± 1.64	3.28 ± 1.33
Interstitial lung disease	5.60 ± 0.87	5.74 ± 0.70	5.69 ± 0.72	5.66 ± 1.04	3.98 ± 1.56	4.69 ± 1.31
Cardiac arrhythmias	4.25 ± 1.61	4.88 ± 1.48	5.19 ± 1.19	5.31 ± 1.38	2.05 ± 1.04	2.16 ± 1.44
Cardiogenic shock	4.55 ± 1.20	4.76 ± 1.23	4.76 ± 1.19	4.97 ± 1.20	3.00 ± 1.23	2.88 ± 1.19
Rheumatic fever	5.00 ± 1.13	5.51 ± 0.98	5.50 ± 0.99	5.50 ± 0.95	3.02 ± 1.41	2.91 ± 1.15
Smoking	5.63 ± 0.71	5.37 ± 1.34	5.43 ± 1.25	5.50 ± 1.30	1.86 ± 1.46	2.31 ± 1.66
Tuberculosis	5.40 ± 0.84	5.49 ± 0.88	5.14 ± 1.20	5.34 ± 1.21	3.45 ± 1.66	3.44 ± 1.50

Data are presented as means ± standard deviations. COPD, chronic obstructive pulmonary disease.

### Impact of the design of the data collection tool on thentest ratings (summer 2012)

**Figure 4:**
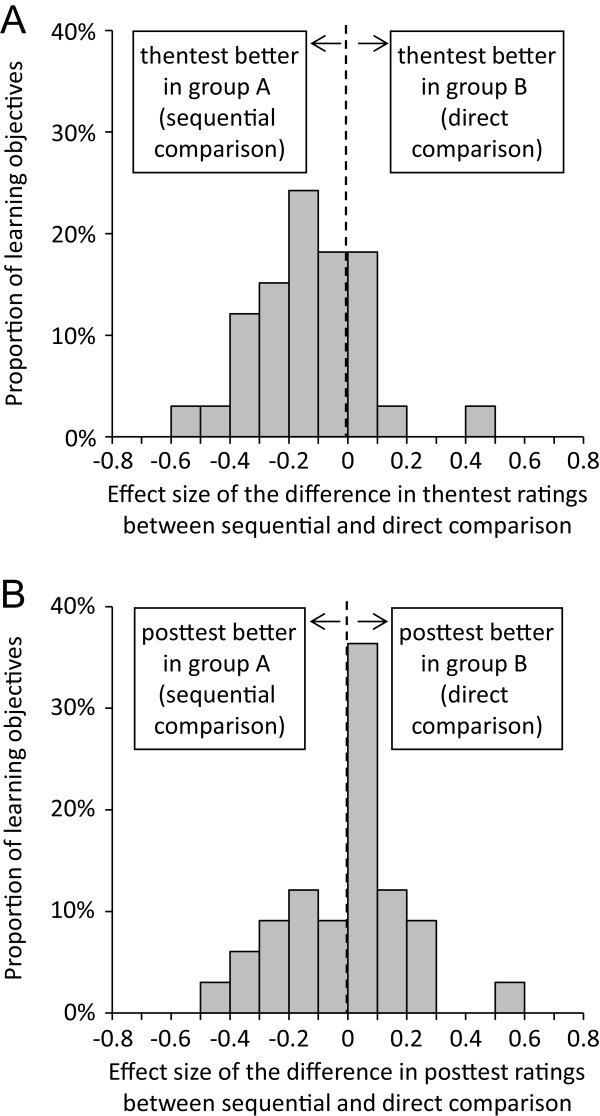
Impact of the design of the data collection tool on (A) thentest and (B) posttest ratings for 33 learning objectives.

**Figure 5:**
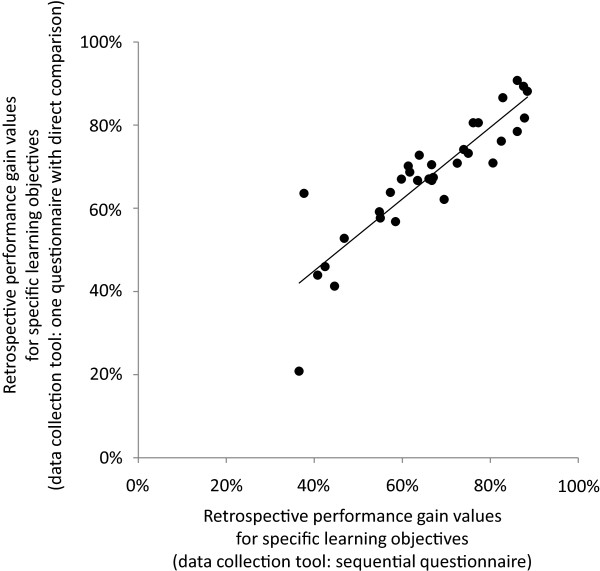
Correlation of retrospective performance gain values between group A (sequential data collection) and group B (direct comparison; r = 0.890; p < 0.001).

In group A (sequential data collection), mean thentest ratings ranged from 2.85 to 5.69; in group B (direct comparison), ratings ranged from 3.63 to 5.66. Effect sizes of the differences between thentest ratings for each of the 33 learning objectives are displayed in Figure 4A. They ranged from -0.55 to 0.42 and were negative for 25 out of 33 learning objectives (mean effect size -0.14, indicating a small effect).In group A, mean posttest ratings ranged from 1.21 to 3.98; in group B, ratings ranged from 1.31 to 4.69. Effect sizes of the differences between posttest ratings for each of the 33 learning objectives are displayed in Figure 4B. They ranged from -0.50 to 0.58 and were negative for 13 out of 33 learning objectives (mean effect size -0.01, indicating almost no effect).Retrospective performance gain was generally higher than in the summer 2011 cohort: In group A, values ranged from 36.6% to 88.4% (mean: 66.0%). Very similar values were found in group B (range: 20.8% to 90.7%; mean: 67.4%). There was a strong and significant correlation between retrospective performance gain values derived from the two different data collection tools (r = 0.890; p < 0.001; see Figure 5). In summary, our hypothesis (b) was not strongly supported by the data.

## Discussion

This study provides insight into the impact of different types of bias on the results of our outcome-based evaluation tool. In accordance with the literature on response shift bias, we found thentest self-ratings of student performance to be more pessimistic than true pretest ratings. However, the mean effect size of this difference was below 0.2, and performance gain values derived from posttests and true pretests were very similar to performance gain values derived from posstests and thentests (mean difference: 3.3%). As expected, the design of the data collection tool did not impact on posttest ratings. Since response shift bias can be assumed to have had a similar effect in both groups, the slightly more pessimistic thentest ratings observed in group B (direct comparison) possibly reflect a minor impact of implicit theories of change in that students directly comparing their thentest and posttest ratings might deliberately inflate the difference between the two if they believed to have learned a lot. However, the mean effect size of the difference between thentest ratings on the two forms was small, and there was virtually no impact on performance gain (mean difference: 1.4%). In summary, using thentests instead of true pretests increases performance gain by a small amount (presumably due to response shift bias). Allowing students to directly compare their thentest and posttest ratings leads to a further but smaller increase in performance gain (presumably due to implicit theories of change).

One practical implication of these findings is that collection of true pretest ratings is not required. This could facilitate a substantial reduction of evaluation activities, thus potentially increasing response rates as students at our institution have expressed a preference for fewer evaluations [7]. In addition, the use of only one data collection point obviates the need for matching students who participated in the first data collection to students who participated in the second one. Matching procedures of this kind typically involve asking students to provide identifying information or codes which can be ethically challenging and is also likely to reduce students’ willingness to participate. Based on this study, our outcome-based evaluation tool can be modified to improve its practicability.

### Comparison with previous studies

The effects of response shift on self-assessments has been researched in considerable depth [9,12,13,16,17]. However, many of these studies focussed on fields other than medical education (e.g., quality of life measurement [22]). As response shift and related origins of bias are context-specific [11], these earlier findings cannot be directly transferred to undergraduate medical education. In addition, previous studies mainly considered the impact of different types of bias on singular ratings or change scores. As performance gain is calculated by dividing the difference between pre- and post-ratings by initial performance levels, it represents a novel measure of teaching quality. Knowing the impact of different types of bias on its results will help to interpret evaluation data.

So far, very few studies have addressed the impact of questionnaire format on thentest ratings. In one study on the effectiveness of a parenting skills course, participants were asked to provide posttest and thentest ratings at the end of the teaching intervention. The data collection tool used in that study consisted of one form with all statements listed in the middle. Participants were asked to enter their thentest ratings in a column left to these statements. They were then required to cover the thentest column before completing the posttest column which was on the right side of the statements. Although this prevented participants from actively comparing their thentest and posttest ratings, the authors report a ‘significant tendency to magnify change scores’ [8]. While this interpretation probably refers to the underlying psychological processes rather than a deliberate decision, our study specifically aimed at assessing the effect of allowing students to manipulate their change score. When presented with a questionnaire that allowed them to directly compare their initial and final performance levels, our students tended to provide more pessimistic thentest ratings. No difference was noted between the two groups regarding posttest ratings, suggesting that these were unaffected by the design of the data collection tool.

### Limitations and suggestions for further research

One particular strength of the present study is its randomised design to assess the impact of questionnaire format on thentest ratings. However, the generalisability of our findings is limited by the monocentric nature of the study as data were collected in one particular module at one German medical school. We tried to ensure high content validity of the questionnaires by including items that had undergone multiple revision and had been used in a previous validation study [5]. Since these items were specific to cardiology and pulmonology and more/less advanced students might be more or less susceptible to the types of bias considered, further research involving larger student groups and covering more subject areas seems warranted. Some related issues are discussed in more detail below.

Our data were derived from two relatively large student cohorts, but response rates were moderate. Response rates for studies involving the completion of multiple questionnaires are usually lower than for studies with just one data collection point. In addition, participation rates observed in this study are comparable to those reported by other groups [6]. Considering that similar response rates have been observed in real life evaluation practices in undergraduate medical education [4,23,24], students participating in this study are likely to represent a typical sample participating in evaluation activities. Yet, given the moderate numbers of students in each group in cohort 2, our data need to be interpreted with caution.

While mean percent scores in the summative end-of-module examination were similar in the two cohorts, selection bias favouring more motivated students or overachievers might have occurred. Since we did not have permission from non-participating students to use their data, we cannot comment on the size and direction of this effect. However, it could be hypothesised that including more motivated students might have increased the validity of our findings as motivated students would be more likely to take the study serious and provide more meticulous ratings. If anything, obtaining more accurate ratings should produce an increase in between-group and within-group differences. Yet, effect sizes of the observed differences were small, suggesting that the impact of response shift bias on evaluation results obtained with the outcome-based tool is at best moderate.

We would like to point out that the present study was not designed to assess criterion validity of the outcome-based evaluation tool. An earlier validation study using objectively measured performance gain as the external criterion showed good agreement between the results of the outcome-based tool and actual learning outcome [5]. The fact that mean retrospective performance gain was higher in the second cohort may be taken as indirect evidence of construct validity of the evaluation tool: As teachers were aware of evaluation results obtained in summer 2011, they might have modified their approach to learning objectives that had received low performance gain values, thus improving evaluation outcome in summer 2012. We cannot test this hypothesis as a total of 70 clinical teachers are involved in the cardio-respiratory module, and there is no 1:1 matching of learning objectives to individual teachers.

The magnitude of bias arising from using thentests instead of true pretests to calculate performance gain may be different in more or less advanced students. Thus, further research involving larger student cohorts from different years of undergraduate medical education is required to verify our current finding of a minor impact of questionnaire design on evaluation results. A second area of uncertainty relates to the formula we used to calculate performance gain (see above): Its denominator decreases as initial performance level increases. Accordingly, any shift in pre-ratings will have greater impact on estimated performance gain if initial performance levels are already high (as the denominator approaches zero). Accordingly, the effect of response shift or implicit theories of change on evaluation results should be greater in more advanced studies, leading to an overestimation of actual performance gain. This hypothesis should be tested in future studies.

## Conclusion

Thentest ratings of initial performance levels were slightly more pessimistic than true pretest ratings, indicating some impact of response shift on student self-assessments in undergraduate medical education. When being allowed to directly compare thentest and posttest ratings, students tended to provide even more pessimistic estimates of their initial performance level, possibly reflecting the impact of implicit theories of change. Overall, the impact of these types of bias on estimated performance gain were small, suggesting that one single data collection at the end of a course using direct comparisons of thentest and posttest ratings is sufficient to generate an adequate appraisal of learning outcome. This is likely to increase both the practicability of the tool and, consequently, student response rates.

## Competing interests

The authors declare that they have no competing interest.

## Authors’ contributions

SS helped to design the study, prepared data analysis and contributed to the manuscript draft. SA provided advice on methodology and contributed to the discussion. TR conceived of the study, designed the study, analysed the data and wrote the manuscript. All authors read and approved the final manuscript.

## Authors’ information

SARAH SCHIEKIRKA is a psychologist at Göttingen University Hospital. She is primarily involved in higher education research, especially clinical teaching and evaluation.

SVEN ANDERS works as a consultant in the Department of Legal Medicine at Hamburg University, co-ordinating the department’s teaching activities. He is involved in curricular development and has just completed a two-year study course of Medical Education. Main research areas are forensic pathology, clinical forensic medicine, and medical education.

TOBIAS RAUPACH is a cardiologist at Göttingen University Hospital. He co-ordinates the department’s teaching activities and has helped to develop the institution’s curriculum. His current research focuses on curricular development, evaluation and assessment formats.

## Pre-publication history

The pre-publication history for this paper can be accessed here:

http://www.biomedcentral.com/1472-6920/14/149/prepub
